# *Escherichia coli* Stress, Multi-cellularity, and the Generation of the Quorum Sensing Peptide EDF

**DOI:** 10.33696/genetics.1.002

**Published:** 2022

**Authors:** Isabella Moll, Hanna Engelberg-Kulka

**Affiliations:** 1 Department of Microbiology, Immunobiology and Genetics, Center for Molecular Biology, University of Vienna, Max Perutz Labs, Vienna Biocenter (VBC), Vienna, Austria; 2 Departmen of Microbiology and Molecular Genetics, IMRIC, The Hebrew University Hadassah Medical School, Ein Karem Jerusalem, Israel

## Abstract

Bacterial communication via quorum sensing (QS) molecules, as well as toxin-antitoxin (TA) gene modules located on bacterial chromosomes are well-studied mechanisms. *Escherichia coli mazEF* is a stress-induced TA system mediating cell death requiring a QS extracellular death factor (EDF), the pentapeptide NNWNN. MazF is an endoribonuclease specific for ACA sites. During adverse conditions, the activated MazF generates a stress induced translation machinery, composed of MazF-processed mRNAs and selective ribosomes that specifically translate these processed mRNAs. Moreover, we identified the molecular mechanism underlying the formation of EDF from the *zwf* mRNA that involves distinct steps comprising the activity of MazF, the trans-translation system as well as the protease ClpPX.

Bacterial trans-translation is generally known as a quality control process that rescues stalled translation complexes at the 3’-terminus of non-stop mRNAs. Our results indicate that trans-translation has a similar role in EDF generation from *zwf* mRNA. However, our data reveal that the trans-translation system may also provide a regulatory mechanism to attenuate EDF generation in the single cells. Thereby, the required threshold of EDF molecules is only achieved by the entire bacterial population, as expected for a genuine QS process.

Bacteria communicate via quorum sensing (QS) signaling molecules [1–8]. QS allows bacteria to monitor and quantify the number of their kin present in their environment. By this means, they ensure the modulation of gene expression with respect to population density. Thereby, bacterial populations can behave like a multicellular organism [1–8]. Over the last years, a great deal of attention has also been focused on the abundance of toxin-antitoxin (TA) gene modules located in chromosomes of most bacteria (reviewed in [9,10]. *E. coli mazEF* is the first discovered chromosomal TA module [11]. The sequence-specific endoribonuclease toxin MazF preferentially cleaves single-stranded mRNAs at the 3’ or at the 5’ side of the first A in the ACA sequences [12,13]. When *E. coli* encounters stressful conditions the antitoxin MazE is proteolytically degraded thereby triggering MazF activity [14]. Under such conditions, the active MazF causes the generation of leaderless mRNAs by the removal of the 5’-UTR of some specific mRNAs by cleavage upstream of AUG start codons [15]. In addition, MazF cleaves the 16S rRNA at the decoding center of the ribosome resulting in its 3'-terminal truncations at position 1500 [15]. The removed 43 nucleotides comprise the anti-Shine-Dalgarno (aSD) region, which is essential for translation initiation on canonical mRNAs but is dispensable for the recognition of AUG start codons located close to the mRNA 5’-terminus. Thus, the aSD-deficient ribosomes exclusively translate leaderless mRNAs [15]. Taken together, the concerted action of MazF on ribosomes and mRNA transcripts engenders a stress-induced translation machinery (STM) [15,16], which allows the specific synthesis of a pool of proteins that affects cell viability within the bacterial population [17,18]. Moreover, the post-transcriptional autoregulatory circuit of *mazF* results in cell-to-cell translation and growth heterogeneity in isogenic populations [19,20].

We have also uncovered the first QS peptide in *E. coli* namely the pentapeptide NNWNN, termed extracellular death factor (EDF). As anticipated for a QS molecule, the pentapeptide is taken up by the cells after the concentration of EDF reaches a threshold level in the medium. In the cytoplasm, it binds to the MazE-binding pocket on MazF, thereby stimulating the activation of MazF. In this respect, EDF acts as signaling molecule linking the *mazEF*-mediated stress response to the population density in *E. coli* [21,22]. Our experiments further revealed that when *E. coli* encounters stressful conditions, activation of MazF results in the generation of the QS EDF factor employing the product of the *zwf* gene [21,22], which encodes the 491–amino acids long enzyme glucose-6-phosphate dehydrogenase involved in the central carbon metabolism [23].

More recently, we studied the MazF-dependent mechanisms underlying the generation of the pentapeptide EDF from the *zwf* gene [24]. We uncovered several distinct molecular steps that are surprisingly involved under stress in the generation of EDF. In particular, we showed that under stress the endoribonuclease MazF cleaves the *zwf* mRNA at specific positions (Figure 1). Thereby, a leaderless *zwf* mRNA variant is engendered that is truncated 30 codons after the EDF-encoding region, a length corresponding to the ribosome nascent peptide exit tunnel. As the absence of a stop codon at the 3’-terminus of the truncated zwf* mRNA results in stalling of translating ribosomes, the trans-translation machinery is recruited to release the truncated Zwf protein (Zwf*) equipped with the C-terminal degradation tag encoded by the tmRNA. Consequently, the protease ClpPX is likewise involved in and essential for the maturation of the EDF pentapeptide (Figure 1).

In general, bacterial trans-translation represents a quality control process that recognizes and rescues stalled ribosome complexes at the 3’-terminus of so-called non-stop mRNAs [25–27]. Taken together, our results indicate that trans-translation is essential for the formation of EDF employing the *zwf* gene (Figure 1). Intriguingly, based on our observations we hypothesize that for EDF synthesis the trans-translation system might further provide a negative feedback loop regulating EDF formation (Figure 2). As numerous mRNA molecules are truncated by active MazF, a plethora of stalled ribosome complexes are formed that recruit the majority of trans-translation components. Thus, only very few of those components are available for the generation of EDF. Collectively, our results support the notion that this process results in the attenuation of EDF generation in the single cell (Figure 2). Thereby, the required threshold of EDF molecules is only achieved by the entire bacterial population, as expected for a genuine quorum sensing (QS) process.

## Figures and Tables

**Figure 1:**
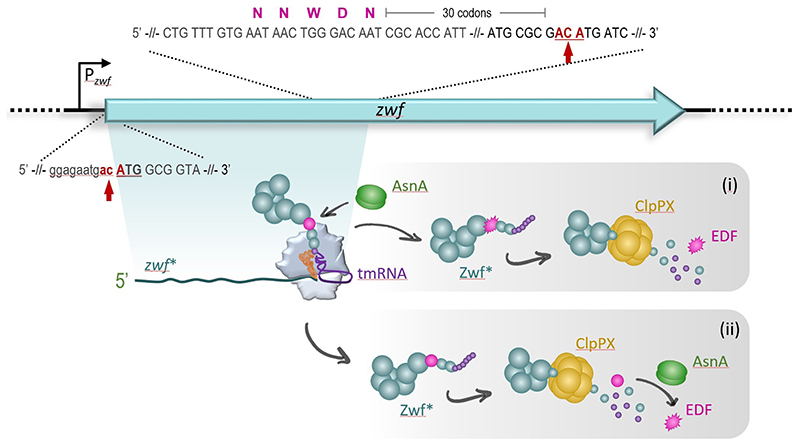
Mechanisms involved in the generation of EDF after activation of MazF during stress. The genetic locus of the *zwf* gene (in cyan) is shown. Active MazF cleaves the *zwf* mRNA at ACA sites (in red, marked by arrows) directly upstream of the start codon (capital letters, underlined) and 30 codons downstream of the codons that code for the pre-EDF pentapeptide NNWDN (in magenta). The resulting leaderless and 3’-terminally truncated zwf* mRNA (cyan) is translated by the specialized ribosomes that stall at the 3’ terminus. Here, the tmRNA (in purple) is essential to resolve the stalled ribosome complex by adding the degradation tag (purple spheres) to the N-terminal part of the Zwf Protein, Zwf* (cyan). The position of the pre-EDF pentapeptide NNWDN is indicated by a magenta sphere. After release the truncated Zwf* protein is subjected to the activity of the ClpP protease. Whether the conversion of the aspartic acid (D) to asparagine (N) by the asparagine synthetase AsnA (green) takes place at the ribosome (i) or after degradation by the ClpPX protease (ii) still remains to be elucidated.

**Figure 2:**
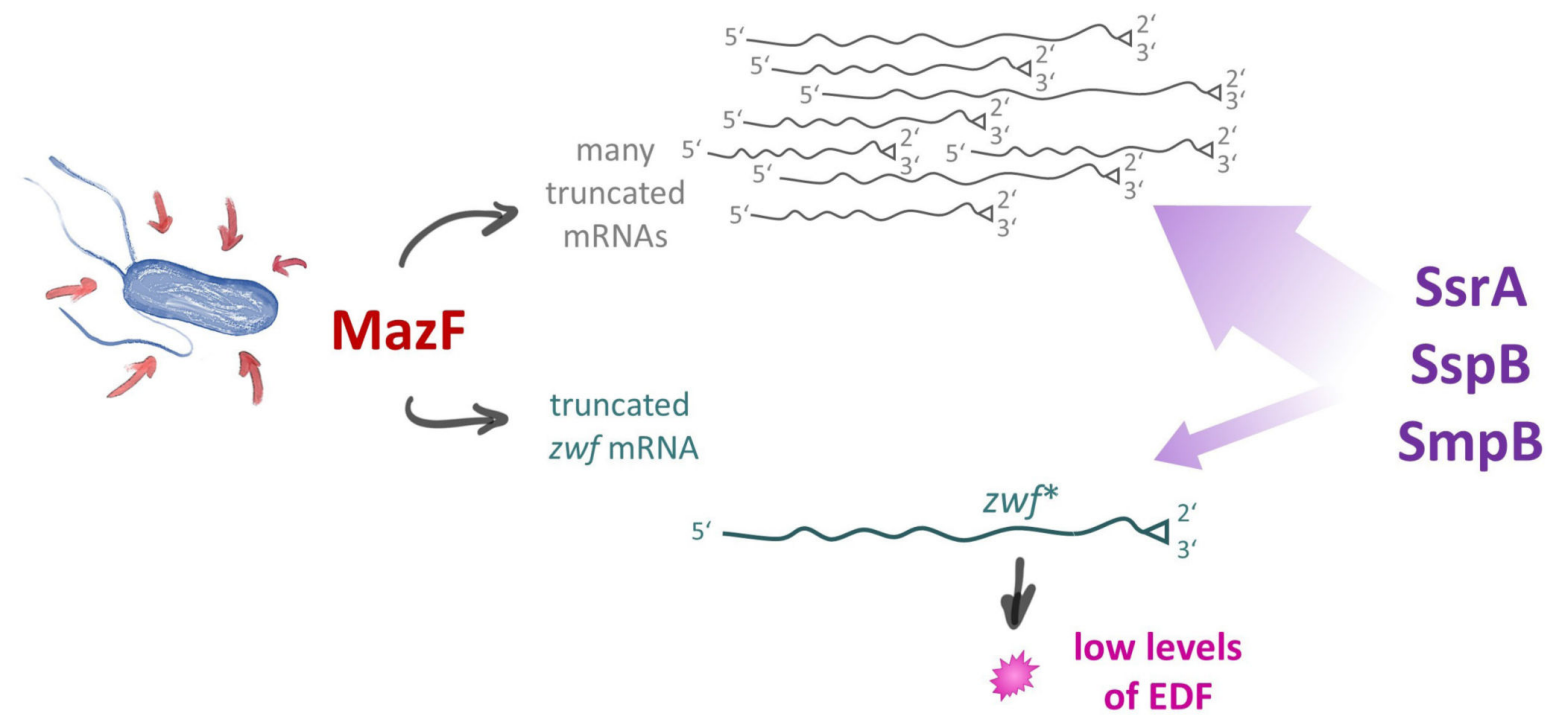
Regulatory circuit ensuring the generation of only low levels of EDF. When *E. coli* cells encounter diverse environmental stress conditions (indicated by red arrows), MazF is activated and cleaves the *zwf* mRNA at the ACA sites indicated in Figure 1 leading to the formation of the zwf* mRNA (in cyan). Besides, MazF activity generates a large number of different truncated mRNAs (in gray). Thus, only a minor fraction of the components of the trans-translational machinery (in purple), namely SsrA, SspB, and SmpB, are available to resolve the stalled ribosome complex on the zwf* mRNA. Consequently, the titration of the trans-translational machinery by stalled complexes on all other truncated mRNAs ensures that only low levels of EDF are generated, as required for a genuine QS molecule.
